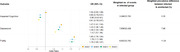# Infections and cognitive function, depression, and frailty: a cross‐sectional study in the Longitudinal Aging Study in India (LASI)

**DOI:** 10.1002/alz.095094

**Published:** 2025-01-09

**Authors:** Georgia R Gore‐Langton, Kathryn E Mansfield, Prithvee Ravi, Arrisonia Doubatty, Suvarna Alladi, Sanjay Kinra, Charlotte Warren‐Gash

**Affiliations:** ^1^ London School of Hygiene and Tropical Medicine, London United Kingdom; ^2^ National Institute of Mental Health and Neuro Sciences, Bengaluru, Karnataka India

## Abstract

**Background:**

Some infections may be associated with poor brain health, but evidence from low and middle‐income countries (LMICs) is limited. Therefore, we aimed to investigate associations between nine infections and cognitive function, depression, and frailty in India.

**Methods:**

We conducted a cross‐sectional study using data from Wave 1 (2017‐2019) of the Longitudinal Aging Study in India (LASI) survey of adults (≥45years) from 35 of India’s 36 states and union territories. Survey data were collected via face‐to‐face interviews and direct health measurements. We investigated the association between nine infections either self‐reported ever (periodontal disease) or in the two years before interview (jaundice/hepatitis, malaria, tuberculosis, typhoid, chikungunya, diarrhoea/gastroenteritis, dengue, urinary tract infection [UTI]), and global cognitive function, depression and frailty. We used survey‐weighted logistic regression to estimate odds ratios comparing impaired cognition, depression, or frailty in people with at least one infection (and each individual infection) to those without infection.

**Results:**

We included 64,682 respondents; median age 59 years (IQR:50‐67), 53.5% female. Accounting for survey weighting, thirty‐five percent (n = 23,783) of respondents reported at least one infection. After adjusting for demographic, social/environmental, lifestyle, and chronic health conditions, we saw evidence of association between having at least one infection and both depression (OR: 1.30 [95%CI: 1.23‐1.36]) and frailty (OR: 1.78 [95%CI: 1.69‐1.87]) (Figure 1). We saw strong associations between jaundice/hepatitis and frailty (OR: 2.20 [95%CI: 1.90‐2.54]), and UTIs and both frailty (OR: 3.08 [95%CI: 2.65‐3.59]) and depression (OR: 1.38 [95%CI:1.19, 1.61]). In contrast, having at least one infection was associated with reduced odds of impaired cognition (OR: 0.81 [95%CI: 0.76‐0.87]).

**Conclusions:**

Our results suggest that infections are associated with increased depression and frailty in adults ≥45 years in India. However, reported infections were associated with better cognition, which may be explained by preferential recall of infections in those with better cognition. Longitudinal studies are needed to investigate a causal link between infections and adverse brain health, and to guide interventions to reduce the burden of impaired cognition, depression, and frailty in India and LMICs more widely.